# Reflections on 10 years of effectiveness-implementation hybrid studies

**DOI:** 10.3389/frhs.2022.1053496

**Published:** 2022-12-08

**Authors:** Geoffrey M. Curran, Sara J. Landes, Sacha A. McBain, Jeffrey M. Pyne, Justin D. Smith, Maria E. Fernandez, David A. Chambers, Brian S. Mittman

**Affiliations:** ^1^Department of Pharmacy Practice, University of Arkansas for Medical Sciences, Little Rock, AR, United States; ^2^Center for Mental Health Outcomes Research, Central Arkansas Veterans Healthcare System, North Little Rock, AR, United States; ^3^Behavioral Health Quality Enhancement Research Initiative (QUERI), Central Arkansas Veterans Healthcare System, Little Rock, AR, United States; ^4^Department of Psychiatry, University of Arkansas for Medical Sciences, Little Rock, AR, United States; ^5^Department of Population Health Sciences, Spencer Fox Eccles School of Medicine at the University of Utah, Salt Lake City, UT, United States; ^6^Center for Health Promotion and Prevention Research, University of Texas, Houston, TX, United States; ^7^Division of Cancer Control and Population Sciences, National Cancer Institute, Rockville, MD, United States; ^8^Department of Research and Evaluation, Kaiser Permanente Southern California, Los Angeles, CA, United States

**Keywords:** implementation science, hybrid studies, research design, cost analysis, health services research, effectiveness-implementation hybrid

## Abstract

This article provides new reflections and recommendations from authors of the initial effectiveness-implementation hybrid study manuscript and additional experts in their conceptualization and application. Given the widespread and continued use of hybrid studies, critical appraisals are necessary. The article offers reflections across five conceptual and methodological areas. It begins with the recommendation to replace the term “design” in favor of “study.” The use of the term “design” and the explicit focus on trial methodology in the original paper created confusion. The essence of hybrid studies is combining research questions concerning intervention effectiveness and implementation within the same study, and this can and should be achieved by applying a full range of research designs. Supporting this recommendation, the article then offers guidance on selecting a hybrid study type based on evidentiary and contextual information and stakeholder concerns/preferences. A series of questions are presented that have been designed to help investigators select the most appropriate hybrid type for their study situation. The article also provides a critique on the hybrid 1-2-3 typology and offers reflections on when and how to use the typology moving forward. Further, the article offers recommendations on research designs that align with each hybrid study type. Lastly, the article offers thoughts on how to integrate costs analyses into hybrid studies.

## Introduction

In 2012, Curran and colleagues (1) proposed hybrid effectiveness-implementation research designs that encouraged combining, in the same study, questions concerning the effectiveness of an intervention with questions about how best to implement it. In addition to the perceived benefit of more rapidly moving toward widespread implementation of interventions in clinical and community settings, the hybrid approach could produce generalizable knowledge on implementation strategies to advance the field. Although hybrids exist on a continuum, Curran et al. proposed three types: Type 1 focuses primarily on testing the effectiveness of an intervention while simultaneously gathering information on implementation factors (e.g., barriers to implementation, potential ways in which to revise the intervention to improve uptake). Type 3 focuses primarily on testing the impact of an implementation strategy (or strategies) on implementation outcomes (e.g., adoption and fidelity of intervention delivery), while simultaneously gathering information on the effectiveness of the intervention. Type 2 has a dual focus, testing both interventions and implementation strategies simultaneously.

As originally proposed, the hybrid effectiveness-implementation design categorizations were applied to experimental *trials*. Randomization was assumed to be happening somewhere, or in the case of some Type 2 studies, perhaps even at multiple levels (e.g., intervention recipients and implementation sites). Further, they were proposed as types of *clinical* trial designs in the sense that the interventions were assumed to be health-related and the places where they were being implemented were healthcare settings of some kind. The intent was not to exclude non-healthcare researchers from applying these designs. The authors (including GMC, JMP, and BSM from this manuscript) were conducting research in healthcare settings [specifically within the United States (US) Department of Veterans Affairs healthcare system] and the main expected audience was other healthcare “services” researchers.

Since the 2012 paper, hybrid effectiveness-implementation designs have become widely adopted within implementation science (2). As of this writing, the original manuscript has been cited over 2000 times. The National Institutes of Health (NIH) in the US and other US funders have explicitly mentioned or requested hybrid studies in funding announcements, and the NIH currently uses the typology to assist with directing proposals to specific grant review panels. Further, the designs are included in many/most national and international training programs (3, 4) in implementation science and interventions research, and Curran and colleagues have provided well over 50 invited presentations/workshops and hundreds of project consultations on hybrids.

With such widespread integration within the field, critical appraisals are necessary. This special issue in *Frontiers in Health Services* is facilitating just that. In recent years, many others have offered extensions and reflections on hybrid designs. For example, in 2018 and 2019 Landsverk et al. (2) and Landes et al. (5) published reflections and novel recommendations for applying hybrid designs. Kemp et al. (6) offered an extension of the typology to more explicitly include context as an independent variable. Chinman et al. (7) provided ways in which hybrid designs could be used in service of research focusing on reducing disparities. Pearson et al. (8) and Wolfenden et al. (8, 9) have written extensively on applications of hybrid studies in small-scale feasibility and pilot studies as well as full-scale RCTs, and Johnson et al. (10) discussed applications of hybrid studies in psychotherapy research.

The current manuscript provides new reflections and recommendations from original authors of the 2012 manuscript (GMC, JMP, and BSM) with additional experts in the conceptualization and application of hybrid designs (SJL, SAM, JDS, MEF, and DAC). The article begins with the recommendation to replace the term “design” in favor of “study.” It is clear that many users are applying hybrids in non-trial designs, and indeed it is possible to conduct a “hybrid study,” which allows the researcher to answer questions about intervention effectiveness and implementation in the same study, using a wide range of research designs. Hence, an effectiveness-implementation hybrid does not require a specific design, but is, instead, a type of study. Supporting this recommendation, the article next presents guidance for selecting the type of hybrid study to conduct based on a range of evidentiary and contextual information and stakeholder concerns and preferences. The article also offers new thinking on the distinctions between the hybrid types, and perhaps most importantly, presents a series of questions designed to help investigators select the most appropriate hybrid type for a given study. The article also examines the basic hybrid type 1-2-3 typology and offers reflections on when and how to refine and use the typology moving forward. For example, the current typology is not adequately reflective of intervention development and research approaches within public health and health promotion/prevention research. Of note, this article focuses on healthcare and health promotion/prevention interventions given that hybrids were developed in the healthcare space, and this is the focus of the authors' work. Hybrids can be used in any evaluation of interventions and implementation outcomes (e.g., education, criminal justice). After a discussion on how to integrate costs analyses into hybrid studies, the article suggests areas for future thinking and writing on effectiveness-implementation hybrid approaches.

These issues are discussed below as responses to questions. Indeed, they represent some of the most *frequently asked questions* posed to the authors at workshops, consultations, and presentations on hybrids.

## Reflections and recommendations

### Are hybrids really “designs”?

As noted above, hybrid designs as originally proposed focused on the design of trials, with explicit attention to *testing* intervention/practices and/or implementation strategies and where and how to consider randomization. At the time, the authors considered proposing additional types or variations, for example, a separate type for observational studies or more explicit discussion of applications for “pilot” studies. In the interests of clarity and practicality, the authors decided to focus the initial typology on trial designs. They noted that the typology was an initial effort which they hoped would “stimulate further thinking and to encourage new design combinations” (1) (page 225). In a later discussion on hybrid designs, Landsverk et al. (2) referred to the originally proposed designs as “ideal types” in the Weberian sense (11), arguing that they were a constructed ideal used to approximate a more complex reality. Thus, they were not intended to cover the universe of possibilities for combining intervention effectiveness and implementation research questions, but to serve as guideposts.

Unfortunately, use of the term “designs” and the explicit focus on trial methodology in the original paper created confusion for many researchers when considering, creating, and ultimately conducting a hybrid study. While the originally-proposed hybrid designs are consistent with Hwang et al.'s (12) recently published definition of research design—“as the planned set of procedures to: (a) select subjects for study; (b) assign subjects to (or observe their natural) conditions; and (c) assess before, during, and after assignment in the conduct of the study” (p. 160)—they clash with the more routinely understood and applied conceptions of research design, e.g., “experimental,” “quasi-experimental,” and “observational” designs (13) or Brown et al.'s (14) typology of “within,” “between,” and “within and between” site designs. Indeed, in the many lectures, consultations, and workshops on hybrids conducted over the years by the authors, a frequent line of questioning centered on whether it was “OK” for hybrid studies to be used in non-trial research designs. The answer was always “yes.” The fundamental purpose of the hybrid framework is to guide selection of study aims, and specifically to determine whether a study should focus primarily on clinical effectiveness while exploring implementation-related factors (type 1), or primarily on implementation effectiveness while measuring clinical effectiveness (type 3), or both (type 2). These decisions are related specifically to study aims, and secondarily to the types of data (specifically outcomes) collected and analyzed, rather than questions of study design. As a result of: (1) the confusion related to the term “designs,” (2) the widespread conduct of hybrid studies employing non-trial designs, and 3) the fact that even after deciding to conduct a hybrid study, a researcher still has to articulate a specific research design, we now recommend dropping the terminology of “hybrid design” and replacing with “hybrid study.”

The essence of a hybrid *study* is that research questions about an intervention's effectiveness and its implementation are contained with the same study. Specific research *designs* are needed to test or evaluate effectiveness and implementation and are guided by the research questions. While specific research designs are not necessarily tied to a hybrid study type, different research designs have more or less utility for each hybrid study type (discussed later).

The hybrid 1-2-3 typology serves to indicate the relative level of emphasis of the effectiveness- and implementation-focused aims or questions within a specific study. In a hybrid type 1 study, the main research question(s), and hence primary outcome(s), are about the performance of an intervention on its target outcomes, e.g., symptoms, functioning, and/or behaviors of individuals or environmental conditions (that contribute behaviors or health outcomes). In a hybrid type 3 study, the main research question(s) and primary outcome(s) are about the performance of an implementation strategy or strategies used to deliver the intervention and the impact of those strategies on an intervention's reach, adoption, fidelity, and/or other implementation outcomes. In a hybrid type 2 study, there are research questions surrounding the performance of both an intervention and implementation strategies, and hence, co-primary outcomes are usually specified.

We recommend authors explicitly note the research design being used and the hybrid type, e.g., “a parallel cluster randomized hybrid type 3 effectiveness-implementation study.” Providing both the research design and the hybrid type is a succinct and informative label for readers and reviewers. Explicitly stating and using the hybrid 1-2-3 study typology language (as opposed to generic labeling as a “hybrid”) serves as an indication of the relative emphasis of the study's intervention- and implementation-focused research questions. We also recommend that authors give an indication of the primary and secondary outcome measures. Achieving clarity on these issues and being explicit about them in research applications and publications will benefit the review process for grant submissions; the planning, execution, and dissemination of the research itself; and facilitate improved documentation and evaluation of the use of hybrid approaches.

### Which hybrid type should I use?

Recently, co-authors GMC, SJL, and JDS developed a tool to use during workshops on hybrid studies that facilitates the selection of a hybrid type. The tool is based on our experiences providing many consultations with numerous investigators interested in conducting hybrid studies. Many investigators knew they wanted to address both intervention effectiveness and implementation questions in the same study, but they were often unsure about which hybrid type made the most sense in their situation. Over time, we came to focus on a series of factors that we feel are critical to selecting a hybrid type for a specific study. The critical issues are: (1) the level of the evidence of the intervention, (2) the extent to which the intervention is expected to be adapted (for context, population, or both), (3) the extent of knowledge about implementation determinants (i.e., barriers/facilitators to implementation), and (4) the extent to which implementation strategies are “ready” to be evaluated. These issues are set against the backdrop for each hybrid type around what are the key advances in knowledge that will come from this study, in our understanding of both intervention and implementation effectiveness.

**Figure 1:**
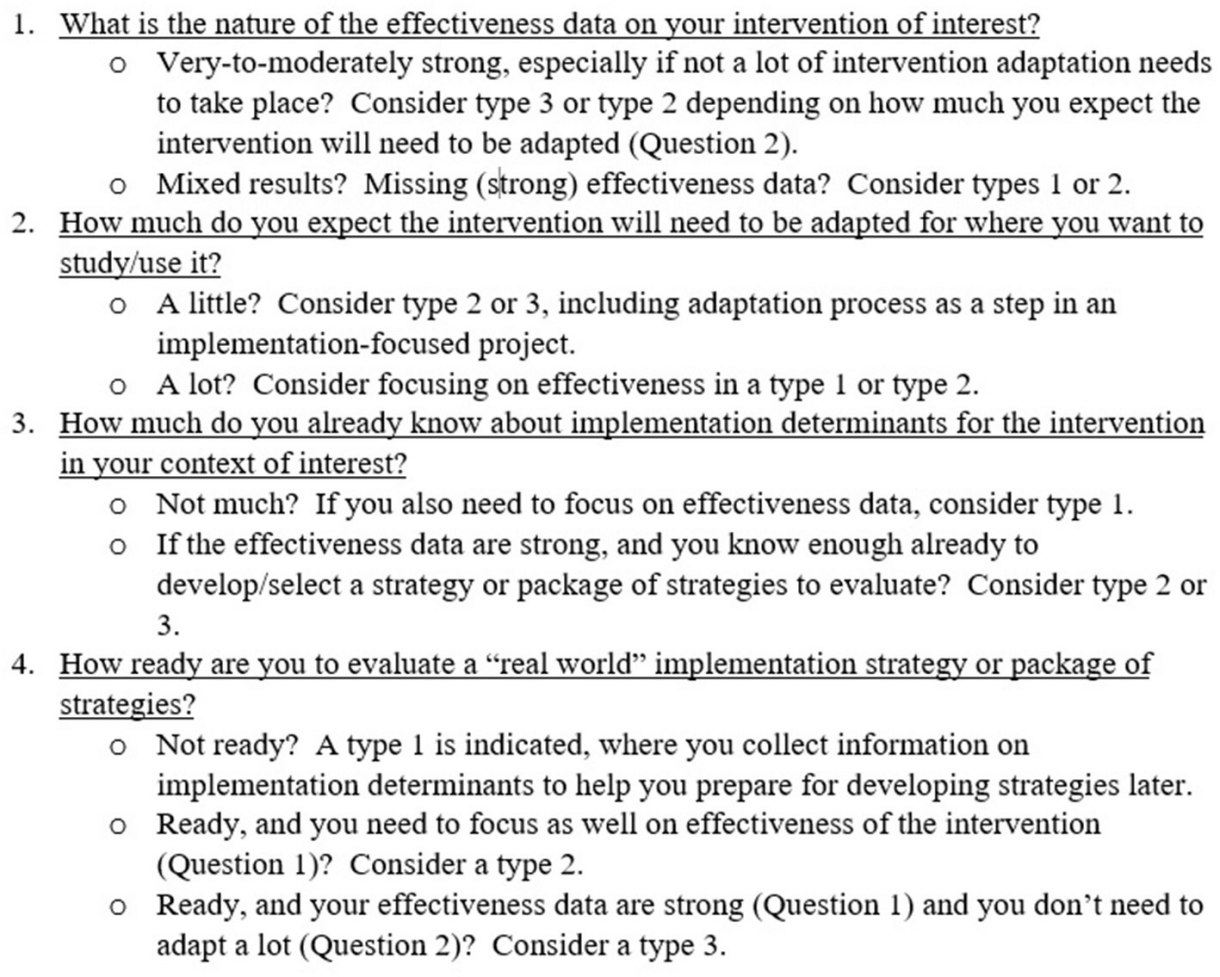
Four questions to consider when selecting a hybrid study type.

Presented in Figure 1, the tool is a series of four questions designed to elicit information on the critical issues noted above relating to an intervention and the relevant implementation context. Depending on the answers, a hybrid type is indicated. The indications in most cases, however, are not absolute. Recommendations usually are to *consider* a certain hybrid type or another based on subjective appraisals of the critical evidence and/or contextual information. The questions are intended to spur reflection and help guide researchers to a hybrid selection that best matches their appraisals. The “final” indication of a hybrid type is based collectively on the answers to the questions. The tool is also useful in determining when a hybrid study is not indicated (also see discussion of this point below).

Question 1 focuses on the strength of effectiveness data that exists currently about the intervention of interest. The presence of strong data in support of an intervention's effectiveness indicates a focus on hybrid types 3 or 2, depending on the extent to which the intervention might need to be adapted (not at all, type 3 is indicated; some adaptation, consider type 2; major adaptation, consider type 1). The relative lack of effectiveness data, or mixed data on effectiveness, indicates a focus on hybrid type 1 or 2, depending on how much is known about implementation determinants. Question 2 focuses on the expected degree of adaptation required to permit use of the intervention for use with a new population, context, or behavioral target (15, 16). Little to no adaption necessary combined with solid effectiveness data on the intervention (Question 1) would indicate a focus on hybrid type 3 or 2. A higher degree of required adaptation would indicate types 1 or 2. Question 3 focuses on current knowledge about the implementation context. If little is known about potential determinants of implementation of an intervention, and there is a strong need for additional effectiveness data (Question 1), a hybrid type 1 is strongly indicated. If enough is known about implementation determinants to be able to select and tailor (17) implementation strategies (see Question 4), a hybrid type 2 or 3 approach is indicated, depending on the existing level of the effectiveness evidence for the intervention (Question 1). Question 4 focuses on readiness to test or evaluate implementation strategies. In the most basic sense, readiness is indicated when investigators have developed or selected the implementation strategies they wish to deploy and evaluate. From an implementation science perspective, such readiness is optimized when implementation strategies have been developed in partnership with relevant key partners (18, 19) and matched to known implementation determinants (20–22). Further, the extent of evidence supporting the use of specific implementation strategies should also be considered. Readiness is subjective, however: the decision to move forward with an evaluation of implementation strategies can be influenced by many factors, including stakeholder input, system needs, and contextual influences (e.g., health emergencies). From the standpoint of selecting a hybrid study type, the decision to evaluate strategies (and therefore “ready”) is the key factor, regardless of how the strategies were selected/developed or how the decision itself was made.

In addition to considering these questions, decision-making on a hybrid study type should also take into account recent clarifications and recommendations by Landes et al. (5), e.g., a type 2 necessitates deployment of implementation strategies developed and hypothesized to be feasible and impactful in real-word settings (a clear distinction from type 1). Further, investigators should consider the extent to which the intervention and implementation strategies are/should be “controlled” and deployed by study personnel vs. personnel in the settings in which the study takes place. For the intervention, greater investigator control will be more common in type 1 and 2 studies and less common in type 3 studies. Within the US Department of Veterans Affairs, funding for hybrid type 3 studies *requires* that the intervention is delivered and paid for by local sites. Likewise, implementation strategies should be delivered by or incorporated into the system or practice whenever possible. Each of these elements of real-world conditions is important to maximize the effectiveness, pragmatic nature of the study, and hence of the resulting findings.

As in many decisions involved in developing a study protocol, the final choice of hybrid type will likely be a balance between what is optimal and what is feasible. There are nuances that arise in the responses to some of the questions as well as pragmatic issues that may influence a researcher's decision. These may include the primary purpose of the study, the number of individuals and sites available for the study, the research design chosen, the primary and secondary research questions, the urgency of results, and the needs of stakeholders. The needs of stakeholders could include, as noted in the original manuscript, opportunities to leverage “momentum” within a healthcare or public health system in favor of rolling out interventions with an incomplete evidence base (e.g., during a health crisis), which would support use of hybrid type 2 and 3 approaches. These considerations must be balanced against the potential harms in moving forward too quickly to implement an intervention with only limited data regarding benefits and harms, and hence requires an assessment of potential harms of the intervention.

The question of potential harms highlights the importance of recognizing the many situations when a hybrid study is not indicated. For example, if an intervention has not yet been established as safe, it could be “too soon” for a hybrid type 1 study, and certainly too soon for a type 2 or 3. On the other end of the spectrum, if an intervention needs no further evaluation (e.g., a highly effective vaccine with robust evidence across multiple settings and populations), collecting further effectiveness evidence through a hybrid type 3 is not needed, and a pure implementation study is indicated with a sole focus on evaluating implementation strategy effectiveness. Further, studies that focus on implementation determinants alone do not need to be a hybrid study because intervention effectiveness is not at issue. Lane-Fall et al. (23) also note the resource intensity of hybrid studies and recommend they should only be undertaken by experienced research teams. At the same time, many in the field currently recommend exploring “implementability” of interventions very early in their development (24, 25) (or adaptation), and there are numerous published hybrid type 1 “pilot” studies (8, 26, 27) indicating a recognition of the value of considering implementation factors very early in intervention development. Given ongoing evolution in this area of research, the authors recommend that investigators justify their rationale when a hybrid study is selected or when considered but not selected.

### Which research design should I use for my hybrid type?

Similar to choosing the hybrid type, the choice of research design is first and foremost guided by the research questions, with considerations of feasibility and investigator and site preferences also important. While research design and hybrid type decisions are not inextricably coupled (i.e., a specific research design does not automatically indicate a hybrid type and vice versa), hybrid types lend themselves to certain research designs by the nature of the associated research questions. Type 1 hybrids most commonly use participant-level randomized controlled trial designs as an internally valid test of the effectiveness of the intervention vs. a control condition (no intervention, usual care, waitlist) is of primary interest. However, cluster randomized and stepped wedge trial designs can also be used for type 1 hybrid studies. With the focus on implementation strategies being co-primary or primary in type 2 and type 3 hybrids respectively, associated research designs tend to focus on meaningful units in the delivery system aligned with the implementation strategy under investigation. For example, a fidelity monitoring strategy for individual clinicians could be tested with a clinician-level randomized design. However, if the fidelity monitoring strategy requires a supervisor, team, and/or health information system that cannot be separated by individual clinician, a cluster randomized design at a higher level would be indicated to maintain internal validity and avoid contamination. Thus, many research designs can be used with type 2 and type 3 hybrids with cluster randomized designs (parallel, stepped wedge, factorial, adaptive) being common.

As noted by Hwang et al. (12), with type 3 designs being most commonly cluster randomized trials, this can result in a small number of units, comparable to similar questions in pure implementation studies. This often raises questions of statistical power in hybrid types. The authors suggest that type 1 trials be powered on participant-level health outcomes; power for implementation outcomes is rarely considered or indicated, and, in many cases, common implementation outcomes such as reach and adoption are often not even measured in type 1 hybrids. Type 2 hybrids could be powered on the health outcome, the implementation outcome, or both, depending on the research design and number of clusters. Type 2 hybrids beg for dual randomized research designs in which units are assigned to both an intervention and an implementation strategy (28), but for various reasons (e.g., feasibility and resources necessary), these are uncommon. Thus, researchers often need to decide on which outcome to power a type 2. It is our observation that most hybrid type 2 studies are either—(1) a person-level randomized study where the implementation strategies are used universally (essentially a pilot study of the strategies supporting the intervention effectiveness trial), or (2) a “single arm” study of both an intervention and implementation approach in a pre-post configuration. Although in Type 2 and 3 hybrids, implementation outcomes are typically measured at the unit level, in some cases, individual (patient or community) level data is used as a proxy for assessing implementation outcomes. For example, the proportion of individuals who received a preventative health screen in a particular clinic can provide information about intervention “penetration” and “fidelity of implementation.”

Type 3 hybrids are necessarily powered to detect effects and differences on one or more implementation outcomes, most often at the level of site, implementer, system, or other unit (e.g., county, state). Depending on the number of sites or clusters, and the reliability of the health outcome measure, many type 3 study research designs will be powered (or overpowered) to detect effects on health outcomes. However, researchers should bear in mind that the number of clusters drives power calculations far more than the number of participants within the cluster or overall study (29).

In this section we have thus far discussed randomized and quasi-experimental designs. However, the hybrid typology can and has been applied to observational designs (30), non-randomized trials (31), and designs more commonly used in formative and pilot work (pre-post/repeated measures within-group, interrupted time-series, etc.). Formative research designs are more likely to be type 1 or type 3 due to limited scope, time, and resources. Observational designs and non-randomized trials can be applied in any hybrid study type but threats to internal validity, reliability of outcome measures, control over the exposure variable (if applicable), and other issues (e.g., sample size) should be considered and used in the justification for choice of research design. We recognize that randomized trials are not always possible or desirable, especially within the context of studying implementation in the “real-world.” Well-designed observational, non-randomized, or “roll-out” studies (14) are of great utility, particularly in the roll-out of public health and community programs. We advocate for using the research/evaluation approach that best suits the needs of investigators and their partners (e.g., health systems, policy makers, communities) and aligns with the research questions. Research designs (not so much hybrid type) must be selected in close partnership with the preferences and capacity of participating entities and implementation partners. Implementation researchers have moral, ethical, and oftentimes legal obligations to ensure that communities and partner sites are not disadvantaged by participation. This issue is underscored when working with communities experiencing health disparities and when health equity is considered (32).

### What are some challenges of the hybrid 1-2-3 typology?

The type 1, 2, and 3 heuristic in and of itself causes challenges for selection and specification of hybrids as the relative focus on effectiveness and implementation exist on a continuum. The three-type typology is a useful shorthand for communicating the intent of the researchers but can fail to capture nuances of the study when a discrete choice of type is necessitated but not necessarily perfectly aligned. Thus, the authors recommend providing a clear rationale and justification for the hybrid type selected that includes reasons for *not* selecting a different hybrid type.

In public health fields such as health promotion and community health that rely on evidence of the effectiveness of interventions tested in real-world settings, efficacy studies often do not precede effectiveness studies. In fact, many studies are designed as evaluations of either existing efforts or newly designed interventions that respond to urgent needs in the community and are evaluated (for the first time) as they are implemented, thus never passing through an efficacy stage. Other research agendas bypass efficacy studies because the value of their findings, given the significant artificiality of features of efficacy research, lead to the conclusion that such studies offer insufficient benefits for the required investment of time and effort. Indeed, because tightly controlled efficacy studies often sacrifice the external validity needed to rapidly scale up implementation efforts, some authors (33) suggest questioning the assumption that effectiveness research logically follows from successful efficacy research. Additionally, since there are often substantial innate differences between interventions tested in efficacy vs. effectiveness studies, particularly related to the way the intervention was delivered (implemented), even when there is good evidence of intervention effects based on efficacy studies, these may not hold when the intervention is delivered and evaluated in real-world settings (thus requiring a hybrid type 2 approach insofar as implementation effects on intervention outcomes are significant and should not be left unexamined).

Most public health/health promotion interventions rely on implementation strategies that are designed to be delivered by practice staff (rather than controlled by researchers) even during the initial evaluation phase. The implementation strategies, in this case, are closely linked with the intervention and are planned during the intervention study design phase, often by a community-engaged planning group that includes adapters and implementers who provide guidance for both the design of the intervention itself (to ensure that it is feasible, acceptable, and effective in their settings) and for the strategies to deliver the intervention in real-world settings. Since hybrid type 1 studies can use investigator-controlled interventions and implementation strategies while type 2 studies should include strategies that are feasible in the real world, many public health/health promotion intervention evaluations begin with a type 2 hybrid.

While there are certainly pitfalls of skipping the efficacy phase, a major advantage is that in real-world studies they can evaluate the effectiveness of interventions as well as their practicality and context. Indeed, much clinical research is not generalizable to non-research settings often because of a failure to document and attend to contextual factors influencing effectiveness.

As mentioned above, the perspectives of all interested parties should be considered when determining the major goals of an evaluation research study and the most appropriate hybrid study type and design. There are several tools that can help researchers determine the extent that their planned study aligns with stated goals. These include the expanded CONSORT figure (34) that helps researchers summarize program participation and representativeness across different settings, staffing structures, and patients/community members. PRECIS-2 (35) and PRECIS-2-PS (36) are other tools that can help researchers make design decisions consistent with the intended purpose of their trial across nine domains—eligibility criteria, recruitment, setting, organization, flexibility (delivery), flexibility (adherence), follow-up, primary outcome, and primary analysis to support decisions about research designs given how the results will be used. Such approaches improve generalizability and potential for scale up (37). Relatedly, existing reporting guidelines can be used with hybrid studies to improve rigor and transparency. While the research design should use the appropriate reporting guidance [e.g., CONSORT (38), a CONSORT extension (39, 40), STROBE (41)], which can be found on the Equator Network website (https://www.equator-network.org/), the format of the Standards for Reporting Implementation Studies (StaRI) statement (42) is well-aligned with all three hybrid study types. When well-defined implementation strategies are being tested or evaluated in a type 2 or 3 hybrid study, investigators might wish to use the template for intervention description and replication (TIDieR) checklist and guide (43).

### How is cost analysis conducted in hybrid approaches?

Examining the costs of delivering an intervention has been a part of healthcare intervention studies for decades (44). A common approach to these economic evaluations is a cost-effectiveness analysis (CEA) comparing the incremental cost and effectiveness of an intervention to usual care in the form of a cost-effectiveness ratio (CER) (45). The details of these analyses are beyond the scope of this paper, but a commonly used patient-level effectiveness outcome is a generic quality-adjusted life year or QALY. A commonly accepted threshold for considering an intervention cost-effective is $100,000 to $15,000 per QALY (46). With increased attention on how best to accelerate and sustain uptake of evidence-based practices in healthcare, there is now increasing attention being paid to the cost of implementation strategies (47). The cost of the implementation strategy (or strategies) needed for uptake and sustainment is critical for presenting an accurate “business case” to decision-makers for implementing and disseminating evidence-based practices.

CEAs can be conducted on a hybrid type 1, 2, or 3 study but are perhaps most informative for a hybrid type 2 or 3 study where a clearly operationalized implementation strategy is being tested. Procedures for costing implementation strategies can be time intensive and need to balance the precision and accuracy of implementation cost data against respondent burden for collecting these data (48, 49). The implications of adding implementation cost to CEAs will be illustrated by two examples. The first example is an intervention that is both more effective than usual care and less expensive (saves money compared to usual care) but requires a more expensive implementation strategy to accelerate uptake and sustainment. Even with the added expense of a more costly implementation strategy the overall CER could still be below the threshold for cost effectiveness. At the other extreme, an intervention could be more effective and more expensive than usual care. In this case a less expensive implementation strategy may be needed to keep the overall CER below the threshold for cost effectiveness.

A CEA could also be performed for an implementation strategy (or strategies) alone. In this case the CER would be the cost associated with the implementation strategy (or strategy combinations/ bundles) divided by an implementation outcome of interest (e.g., fidelity, adoption, penetration). A league table similar to those that rank interventions from most cost effective (lower CER) to least cost-effective (higher CER) could be developed for implementation strategies. For example, starting with a table listing implementation strategies, columns could be added for cost and cost per implementation outcome. Such a table could inform the choice of implementation strategy for future studies.

Several considerations are important when assessing the cost of implementation activities. First, it is important to identify the perspective with which the primary decision-maker is going to view the implementation cost data. Perspectives to consider include societal, payer, organizational, facility, provider, and patient (50). The decision-makers' perspective will determine which costs to include and the choice of outcome measure. Second, prospective measurement of resources, including staff time spent on implementation activities, is superior to retrospective measurement, suggesting the need to plan for implementation time and cost measurement early in the study design process to allow for prospective data collection. Third, time spent on implementation activities will vary by site (51). Therefore, the implementation activity time at one site may not apply to all sites. Fourth, qualitative data collection may be important for capturing information that will inform the interpretation of the economic evaluation (e.g., context for understanding site-level variation in implementation activity and stakeholder interpretation of results) (52).

## Conclusion

The proliferation of hybrid studies highlights their potential to facilitate successful quality and outcome improvement in numerous areas of healthcare, health promotion, and health policy, and speed the translation of research findings into routine practice. This reflection paper was intended to bring greater clarity to the selection and application of hybrid studies.

The future of hybrid studies should be informed by reflections from Beidas et al. in a recent commentary (32). They highlighted the potential threats to progress in the field, including the potential for limited impact on population health and health equity, a re-creation of the research-to-practice gap, the inaccessibility of implementation science, and the misalignment with implementation partners' needs and priorities. Several of Beidas et al.'s proposed solutions align with the core tenets of hybrid studies discussed here. Hybrid studies offer opportunities to promote health equity by emphasizing routine inclusion of health outcomes in implementation studies, measurement of the impact of implementation strategies on outcomes (including health equity outcomes), increased focus on the associations between implementation outcomes and health outcomes, and greater collaboration with key partners to bring about pragmatic solutions. However, more work is needed to leverage the potential utility of hybrid studies in these areas. We encourage greater focus and study on the potential utility of hybrid studies in promoting advancement in our field overall and specifically in the areas of health equity and social justice.

## Data availability statement

The original contributions presented in the study are included in the article/supplementary material, further inquiries can be directed to the corresponding author.

## Author contributions

All authors listed have made a substantial, direct, and intellectual contribution to the work and approved it for publication.

## References

[1] Curran GM Bauer M Mittman B Pyne JM Stetler C. Effectiveness-implementation hybrid designs: combining elements of clinical effectiveness and implementation research to enhance public health impact. Med Care. (2012) 50:217–26. 10.1097/MLR.0b013e3182408812 22310560 PMC3731143

[2] Landsverk J Brown CH Smith JD Chamberlain P Curran GM Palinkas L. Design and Analysis in Dissemination and Implementation Research. In: Brownson RC, Colditz GA, Proctor EK editors. Dissemination and Implementation Research in Health: Translating Science to Practice. 2nd ed: Oxford University Press (2017). p. 201–28.

[3] Proctor EK Landsverk J Baumann AA Mittman BS Aarons GA Brownson RC. The implementation research institute: training mental health implementation researchers in the United States. Implement Sci. (2013) 8:105. 10.1186/1748-5908-8-105 24007290 PMC3844451

[4] Schwartz SR Smith JD Hoffmann C Hansoti B Mishra S Means AR. Implementing implementation research: teaching implementation research to HIV researchers. Curr HIV/AIDS Rep. (2021) 18:186–97. 10.1007/s11904-021-00551-4 33709323

[5] Landes SJ McBain SA Curran GM. An introduction to effectiveness-implementation hybrid designs. Psychiatry Res. (2019) 280:112513. 10.1016/j.psychres.2019.112513 31434011 PMC6779135

[6] Kemp CG Wagenaar BH Haroz EE. Expanding hybrid studies for implementation research: intervention, implementation strategy, and context. Front Public Health. (2019) 7:325. 10.3389/fpubh.2019.00325 31781528 PMC6857476

[7] Chinman M Woodward EN Curran GM Hausmann LRM. Harnessing Implementation Science to Increase the Impact of Health Equity Research. Med Care. (2017) 55 Suppl 9 Suppl 2:S16–S23. 10.1097/MLR.0000000000000769 28806362 PMC5639697

[8] Pearson N Naylor PJ Ashe MC Fernandez M Yoong SL Wolfenden L. Guidance for conducting feasibility and pilot studies for implementation trials. Pilot Feasibility Stud. (2020) 6:167. 10.1186/s40814-020-00634-w 33292770 PMC7603668

[9] Wolfenden L Foy R Presseau J Grimshaw JM Ivers NM Powell BJ. Designing and undertaking randomised implementation trials: guide for researchers. BMJ. (2021) 372:m3721. 10.1136/bmj.m3721 33461967 PMC7812444

[10] Johnson AL Ecker AH Fletcher TL Hundt N Kauth MR Martin LA. Increasing the impact of randomized controlled trials: an example of a hybrid effectiveness-implementation design in psychotherapy research. Transl Behav Med. (2020) 10:629–36. 10.1093/tbm/iby116 30476315

[11] Weber M Shils E Finch HA. Max Weber on the Methodology of the Social Sciences Vol 1. Glencoe, IL: Free Press (1949).

[12] Hwang S Birken SA Melvin CL Rohweder CL Smith JD. Designs and methods for implementation research: Advancing the mission of the CTSA program. J Clin Transl Sci. (2020) 4:159–67. 10.1017/cts.2020.16 32695483 PMC7348037

[13] Campbell D Stanley J. Experimental and Quasi-Experimental Designs for Research. Boston, MA: Houghton Mifflin Co. (2010).

[14] Brown CH Curran G Palinkas LA Aarons GA Wells KB Jones L. An Overview of Research and Evaluation Designs for Dissemination and Implementation. Annu Rev Public Health. (2017) 38:1–22. 10.1146/annurev-publhealth-031816-044215 28384085 PMC5384265

[15] Smith JD Berkel C Rudo-Stern J Montaño Z St George SM Prado G. The Family Check-Up 4 Health (FCU4Health): Applying Implementation Science Frameworks to the Process of Adapting an Evidence-Based Parenting Program for Prevention of Pediatric Obesity and Excess Weight Gain in Primary Care. Front Public Health. (2018) 6:293. 10.3389/fpubh.2018.00293 30374436 PMC6196330

[16] Aarons GA Sklar M Mustanski B Benbow N Brown CH. “Scaling-out” evidence-based interventions to new populations or new health care delivery systems. Implement Sci. (2017) 12:111. 10.1186/s13012-017-0640-6 28877746 PMC5588712

[17] Powell BJ Beidas RS Lewis CC Aarons GA McMillen JC Proctor EK. Methods to Improve the Selection and Tailoring of Implementation Strategies. J Behav Health Serv Res. (2017) 44:177–94. 10.1007/s11414-015-9475-6 26289563 PMC4761530

[18] Powell BJ Fernandez ME Williams NJ Aarons GA Beidas RS Lewis CC. Enhancing the impact of implementation strategies in healthcare: a research agenda. Front Public Health. (2019) 7:3. 10.3389/fpubh.2019.00003 30723713 PMC6350272

[19] Dopp AR Parisi KE Munson SA Lyon AR. Aligning implementation and user-centered design strategies to enhance the impact of health services: results from a concept mapping study. Implement Sci Commun. (2020) 1:17. 10.1186/s43058-020-00020-w 32885179 PMC7427975

[20] Baker R Camosso-Stefinovic J Gillies C Shaw EJ Cheater F Flottorp S. Tailored interventions to address determinants of practice. Cochrane Database Syst Rev. (2015) 4:CD005470. 10.1002/14651858.CD005470.pub3 25923419 PMC7271646

[21] Kwok EYL Moodie STF Cunningham BJ Oram Cardy JE. Selecting and tailoring implementation interventions: a concept mapping approach. BMC Health Serv Res. (2020) 20:385. 10.1186/s12913-020-05270-x 32375752 PMC7203846

[22] Knapp AA Carroll AJ Mohanty N Fu E Powell BJ Hamilton A. A stakeholder-driven method for selecting implementation strategies: a case example of pediatric hypertension clinical practice guideline implementation. Implement Sci Commun. (2022) 3:25. 10.1186/s43058-022-00276-4 35256017 PMC8900435

[23] Lane-Fall MB Curran GM Beidas RS. Scoping implementation science for the beginner: locating yourself on the “subway line” of translational research. BMC Med Res Methodol. (2019) 19:133. 10.1186/s12874-019-0783-z 31253099 PMC6599376

[24] Klaic M Kapp S Hudson P Chapman W Denehy L Story D. Implementability of healthcare interventions: an overview of reviews and development of a conceptual framework. Implement Sci. (2022) 17:10. 10.1186/s13012-021-01171-7 35086538 PMC8793098

[25] Kwan BM Brownson RC Glasgow RE Morrato EH Luke DA. Designing for dissemination and sustainability to promote equitable impacts on health. Annu Rev Public Health. (2022) 43:331–53. 10.1146/annurev-publhealth-052220-112457 34982585 PMC9260852

[26] Anton MT Ridings LE Hanson R Davidson T Saunders B Price M. Hybrid type 1 randomized controlled trial of a tablet-based application to improve quality of care in child mental health treatment. Contemp Clin Trials. (2020) 94:106010. 10.1016/j.cct.2020.106010 32320845 PMC7357202

[27] Beidas RS Ahmedani BK Linn KA Marcus SC Johnson C Maye M. Study protocol for a type III hybrid effectiveness-implementation trial of strategies to implement firearm safety promotion as a universal suicide prevention strategy in pediatric primary care. Implement Sci. (2021) 16:89. 10.1186/s13012-021-01154-8 34551811 PMC8456701

[28] Garner BR Gotham HJ Chaple M Martino S Li JHF Roosa MR. The implementation and sustainment facilitation strategy improved implementation effectiveness and intervention effectiveness: results from a cluster-randomized, type 2 hybrid trial. Implement Res Pract. (2020) 1:1–23. 10.1177/2633489520948073 36189179 PMC9523796

[29] Hemming K Taljaard M. Sample size calculations for stepped wedge and cluster randomised trials: a unified approach. J Clin Epidemiol. (2016) 69:137–46. 10.1016/j.jclinepi.2015.08.015 26344808 PMC4687983

[30] Aron DC Lowery J Tseng CL Conlin P Kahwati L. De-implementation of inappropriately tight control (of hypoglycemia) for health: protocol with an example of a research grant application. Implement Sci. (2014) 9:58. 10.1186/1748-5908-9-58 24886315 PMC4046046

[31] Smith MJ Smith JD Jordan N Sherwood K McRobert E Ross B. Virtual reality job interview training in transition services: results of a single-arm, noncontrolled effectiveness-implementation hybrid trial. J Spec Educ Technol. (2021) 36:3–17. 10.1177/0162643420960093 PMC11192452 38911489

[32] Beidas RS Dorsey S Lewis CC Lyon AR Powell BJ Purtle J. Promises and pitfalls in implementation science from the perspective of US-based researchers: learning from a pre-mortem. Implement Sci. (2022) 17:55. 10.1186/s13012-022-01226-3 35964095 PMC9375077

[33] Glasgow RE Lichtenstein E Marcus AC. Why don't we see more translation of health promotion research to practice? Rethinking the efficacy-to-effectiveness transition. Am J Public Health. (2003) 93:1261–7. 10.2105/AJPH.93.8.1261 12893608 PMC1447950

[34] Glasgow RE Huebschmann AG Brownson RC. Expanding the CONSORT figure: increasing transparency in reporting on external validity. Am J Prev Med. (2018) 55:422–30. 10.1016/j.amepre.2018.04.044 30033029

[35] Loudon K Treweek S Sullivan F Donnan P Thorpe KE Zwarenstein M. The PRECIS-2 tool: designing trials that are fit for purpose. BMJ. (2015) 350:h2147. 10.1136/bmj.h2147 25956159

[36] Norton WE Loudon K Chambers DA Zwarenstein M. Designing provider-focused implementation trials with purpose and intent: introducing the PRECIS-2-PS tool. Implement Sci. (2021) 16:7. 10.1186/s13012-020-01075-y 33413489 PMC7791810

[37] Huebschmann AG Leavitt IM Glasgow RE. Making health research matter: a call to increase attention to external validity. Annu Rev Public Health. (2019) 40:45–63. 10.1146/annurev-publhealth-040218-043945 30664836

[38] Schulz KF Altman DG Moher D. CONSORT 2010 statement: Updated guidelines for reporting parallel group randomised trials. J Pharmacol Pharmacother. (2010) 1:100–7. 10.4103/0976-500X.72352 21350618 PMC3043330

[39] Campbell MK Piaggio G Elbourne DR Altman DG. Consort 2010 statement: extension to cluster randomised trials. BMJ. (2012) 345:e5661. 10.1136/bmj.e5661 22951546

[40] Hemming K Taljaard M McKenzie JE Hooper R Copas A Thompson JA. Reporting of stepped wedge cluster randomised trials: extension of the CONSORT 2010 statement with explanation and elaboration. BMJ. (2018) 363:k1614. 10.1136/bmj.k1614 30413417 PMC6225589

[41] Vandenbroucke JP von Elm E Altman DG Gøtzsche PC Mulrow CD Pocock SJ. Strengthening the Reporting of Observational Studies in Epidemiology (STROBE): explanation and elaboration. Ann Intern Med. (2007) 147:W163–194. 10.7326/0003-4819-147-8-200710160-00010-w1 17938389

[42] Pinnock H Barwick M Carpenter CR Eldridge S Grandes G Griffiths CJ. Standards for Reporting Implementation Studies (StaRI) Statement. BMJ. (2017) 356:i6795. 10.1136/bmj.i6795 28264797 PMC5421438

[43] Hoffmann TC Glasziou PP Boutron I Milne R Perera R Moher D. Better reporting of interventions: template for intervention description and replication (TIDieR) checklist and guide. BMJ. (2014) 348:g1687. 10.1136/bmj.g1687 24609605

[44] Gold M Siegel JE Russell LB. Cost-effectiveness in Health and Medicine. 1 ed. New York: Oxford University Press (1996).

[45] Neumann PJ Sanders GD Russell LB Siegel JE Ganiats TG. Cost-Effectiveness in Health and Medicine. 2nd ed. Oxford: Oxford University Press (2016).

[46] Neumann PJ Cohen JT Weinstein MC. Updating cost-effectiveness–the curious resilience of the $50,000-per-QALY threshold. N Engl J Med. (2014) 371:796–7. 10.1056/NEJMp1405158 25162885

[47] Eisman AB Kilbourne AM Dopp AR Saldana L Eisenberg D. Economic evaluation in implementation science: Making the business case for implementation strategies. Psychiatry Res. (2020) 283:112433. 10.1016/j.psychres.2019.06.008 31202612 PMC6898762

[48] Wagner TH Yoon J Jacobs JC So A Kilbourne AM Yu W. Estimating Costs of an Implementation Intervention. Med Decis Making. (2020) 40:959–67. 10.1177/0272989X20960455 33078681

[49] Cidav Z Mandell D Pyne J Beidas R Curran G Marcus S. Pragmatic method for costing implementation strategies using time-driven activity-based costing. Implement Sci. (2020) 15:28. 10.1186/s13012-020-00993-1 32370752 PMC7201568

[50] Eisman AB Quanbeck A Bounthavong M Panattoni L Glasgow RE. Implementation science issues in understanding, collecting, and using cost estimates: a multi-stakeholder perspective. Implement Sci. (2021) 16:75. 10.1186/s13012-021-01143-x 34344411 PMC8330022

[51] Wong ES Rajan S Liu CF Morland LA Pyne JM Simsek-Duran F. Economic costs of implementing evidence-based telemedicine outreach for posttraumatic stress disorder in VA. Implement Res Pract. (2022) 3:26334895221116771. 10.1177/26334895221116771 PMC9924252 37091111

[52] Dopp AR Mundey P Beasley LO Silovsky JF Eisenberg D. Mixed-method approaches to strengthen economic evaluations in implementation research. Implement Sci. (2019) 14:2. 10.1186/s13012-018-0850-6 30635001 PMC6329154

